# Human Ischaemic Cascade Studies Using SH-SY5Y Cells: a Systematic Review and Meta-Analysis

**DOI:** 10.1007/s12975-018-0620-4

**Published:** 2018-03-23

**Authors:** Ye Liu, Emma D. Eaton, Taryn E. Wills, Sarah K. McCann, Ana Antonic, David W. Howells

**Affiliations:** 1 The Florey Institute of Neuroscience and Mental Health, 30 Royal Parade, The University of Melbourne, Melbourne, VIC 3052 Australia; 2 School of Medicine, Faculty of Health, University of Tasmania, Medical Sciences Precinct, 17 Liverpool Street, Hobart, TAS 7000 Australia; 3 Melbourne Brain Centre, Florey Institute of Neuroscience and Mental Health, 245 Burgundy St, Heidelberg, VIC 3084 Australia; 4 Centre for Clinical Brain Sciences, University of Edinburgh, Edinburgh, EH16 4SB UK; 5 Department of Neuroscience, Monash University, Melbourne, VIC 3004 Australia; 6 School of Medicine, Faculty of Health, University of Tasmania, Medical Sciences Precinct, 17 Liverpool Street, Hobart, TAS 7000 Australia

**Keywords:** Human ischaemic cascade, SH-SY5Y cells, Systematic review and meta-analysis, In vitro ischaemia-related injuries, Study quality

## Abstract

Low translational yield for stroke may reflect the focus of discovery science on rodents rather than humans. Just how little is known about human neuronal ischaemic responses is confirmed by systematic review and meta-analysis revealing that data for the most commonly used SH-SY5Y human cells comprises only 84 papers. Oxygen-glucose deprivation, H_2_O_2_, hypoxia, glucose-deprivation and glutamate excitotoxicity yielded − 58, − 61, − 29, − 45 and − 49% injury, respectively, with a dose-response relationship found only for H_2_O_2_ injury (*R*^2^ = 29.29%, *p* < 0.002). Heterogeneity (*I*^2^ = 99.36%, *df* = 132, *p* < 0.0001) was largely attributable to the methods used to detect injury (*R*^2^ = 44.77%, *p* < 0.000) with cell death assays detecting greater injury than survival assays (− 71 vs − 47%, *R*^2^ = 28.64%, *p* < 0.000). Seventy-four percent of publications provided no description of differentiation status, but in the 26% that did, undifferentiated cells were susceptible to greater injury (*R*^2^ = 4.13%, *p* < 0.047). One hundred and sixty-nine interventions improved average survival by 34.67% (*p* < 0.0001). Eighty-eight comparisons using oxygen-glucose deprivation found both benefit and harm, but studies using glutamate and H_2_O_2_ injury reported only improvement. In studies using glucose deprivation, intervention generally worsened outcome. There was insufficient data to rank individual interventions, but of the studies reporting greatest improvement (> 90% effect size), 7/13 were of herbal medicine constituents (24.85% of the intervention dataset). We conclude that surprisingly little is known of the human neuronal response to ischaemic injury, and that the large impact of methodology on outcome indicates that further model validation is required. Lack of evidence for randomisation, blinding or power analysis suggests that the intervention data is at substantial risk of bias.

**Electronic supplementary material:**

The online version of this article (10.1007/s12975-018-0620-4) contains supplementary material, which is available to authorized users.

## Introduction

Translation in the field of stroke neuroprotection has proven to be particularly challenging [1]. In part, this may be because our understanding of human stroke pathophysiology is incomplete, and consequently, we may not have targeted the right cells or the right molecular processes within these cells [2]. At present, our understanding of the processes of the ischaemic cascade comes mainly from experiments in rodent grey matter [3] and remarkably little seems to be known of the human neural ischaemic response.

To begin to systematise our knowledge of human neuron ischaemic responses, we chose to identify the cell types studied within the human dataset because their names are unambiguous and then to select the most frequently used cell types for systematic review and meta-analysis. We used the SWIFT-Review software [4] developed by SCIOME to perform a word frequency analysis to identify the cell types (with the search term “cells”) used within the titles and abstracts of the PubMed component of our initial search (at this time SWIFT only worked directly with PubMed). This search revealed that a much smaller number of papers explicitly identified the cell type they used, and amongst these, human neuroblastoma-derived SH-SY5Y cells are by far the most commonly used human cell type (Table 1). Therefore, it was decided to concentrate on this cell type and to systematically search the literature for studies investigating ischaemia-related injuries in SH-SY5Y cell culture.

**Table 1:** Most frequently used human cell lines in aforementioned PubMed search identified by SWIFT

Human brain cell types used	Abbreviated designators	Document frequency
Human neuroblastoma	SH-SY5Y	139
	SK-N-SH	27
	SK-N-MC	
	IMR-5	8
	IMR-32	
	IMR-90	
Human terato-carcinoma-derived neuron-like cells (Ntera2/D1)	NT2-N	11
Human glioblastoma cells	U87	5
	T98G	2
Human astrocytoma	CCF-STTG1	2
	U373 MG	1
	1321N1	1
Human cortical neurons	HCN-1A	2
Non-brain human cell lines used		
Human umbilical vein endothelial cells	HUVEC	28
Human umbilical cord blood cells derived-mesenchymal stem cells	HUCB-MSC	3
Foetal human neural stem and progenitor cells	fNPCs	2

PubMed search performed in 12 August 2014; SWIFT search performed in May 2016

The ischaemic cascade starts with blood flow and energy supply reduction [5], which subsequently leads to excitotoxicity, oxidative stress, and cell death [6, 7]. Ischaemic injury to cells in vitro is induced by hypoxia [8], glucose deprivation [9] and the combination of oxygen and glucose deprivation (OGD) [10] to model energy deprivation. Application of glutamate/N-methyl-D-aspartate (NMDA) is used to model excitotoxicity [11], and H_2_O_2_ [12] and sodium nitroprusside (SNP) [13] are used to model oxidative stress. Tumour necrosis factor-α (TNF-α) has been given to cause nuclear factor-kappa (NF-κB)-mediated cell damage [14]. Using these injury models, the responses of SH-SY5Y cell lines have been studied to elucidate the roles of small molecules and pathways involving in the ischaemic cascade. These models have also been used as an in vitro platform to test the neuroprotective efficacy of several compounds [12, 15, 16]. Therefore, meta-analysis and meta-regression were conducted to investigate the responses of SH-SY5Y cells to different injury models and interventions.

## Material and Methods

### Identification of Relevant Studies

#### Searching

Three databases, Pubmed, Embase and Web of Science, were searched for the terms “SHSY5Y OR SH-SY5Y OR SH-SY-5Y OR SHSY-5Y OR SH-SY” AND “brain ischemia OR brain ischaemia OR brain ischemic OR brain infarctions OR brain infarction OR cerebral infarction OR cerebral infarctions OR stroke OR ischemic stroke” on 3 January 2017 (the detailed search strategy is reported in Appendix 2).

The citations retrieved were pooled in Endnote 7, and duplicates were identified and removed using the built-in duplicate removal tool. Screening of the titles and abstracts was then performed by two independent reviewers (E.E., T.W./S.M.) who were familiar with preclinical stroke research and able to apply the inclusion and exclusion criteria detailed below. Disagreements were adjudicated by a third person (A.A.) who also worked in the stroke research field. Full texts were then assessed for their eligibility as described below before data extraction and meta-analysis.

#### Definition

A “publication” was defined as a discrete piece of work (excluding abstracts) containing data from one or more experiments, each of which may describe outcomes using multiple experimental readouts. An “individual comparison” was defined as the contrast between readouts in a single injury cohort compared with that in an untreated cohort or a single intervention cohort compared with an appropriate injured control cohort.

For many studies, the sample size and the number of technical replicates were unclear. We believe that in most instances, the authors intended the “*n*” specified to indicate the number of times each experiment had been repeated independently on different days or in different experimental runs. Therefore, we took a pragmatic approach of using the originating authors’ “*n*” to be the sample size applied to each experiment. Because this number was generally so low, we did not adjust the numbers for weight and considered each to be an independent study. This was not the usual approach found in systematic review and meta-analysis, but we felt that we had little choice but to adopt this approach. The percentage of publications where the authors indicated a difference between technical replicates and the number of such replicates were given in the “Results” section.

Also, for the purposes of this study, we have considered the SH-SY5Y cell line to be a single biological entity. “*n*” is not the same as the number of individuals recruited into a clinical trial or the number of animals used in an in vivo experiment, but the number of times a particular experimental contrast has been assessed.

#### Inclusion and Exclusion Criteria

We included controlled studies that quantified cell death or survival as an outcome in SH-SY5Y cells after ischaemia-related injury. This was limited to models of glucose deprivation, hypoxia or combined oxygen glucose deprivation (OGD), oxidative stress imposed by the application of H_2_O_2_ or sodium nitroprusside (SNP), and injury caused by glutamate and TNF-α. Genetically modified SH-SY5Y studies were only included under the aforementioned injury models. To be included, studies needed to report sample sizes, mean of control and injury, standard deviation or standard error, injury duration and interventions. Study authors were contacted via e-mail if these data were absent in the full papers.

We excluded studies without control groups or not reporting any form of cell death/survival readout. We excluded studies reporting non-ischaemic-related injury models such as the Parkinson’s disease model created by the application of MPP+ or 6-OHDA and the Alzheimer disease model created by the application of A-β. Studies where relevance to ischaemic injury was unclear were also excluded from analysis but recorded for future examination. Toxicants targeting the final common pathway of cell death were excluded from analysis but also recorded for future analysis (Staurosporine/STS, Thapsigargin/TG or other cell death inducers). Conference abstracts were excluded.

#### Data Extraction

Outcomes of injury magnitude compared to uninjured cohorts and of interventions compared against injury controls were extracted. Only the dose or time which achieved the best outcome/greatest injury was extracted from dose- or time-dependent curves to reflect the best outcome of one intervention/greatest level of injury. When a publication reported more than one type of cell death or survival readout (such as reporting both MTT and LDH in one study), we considered these to be independent experiments and extracted data for each of these. Where different outcome units or measuring methods were used for a single readout (such as propidium iodide (PI) staining of individual cells counted under the microscope plus staining of PI measured by fluorescence activated cell sorting), we nested them into one group, and the normalized mean differences were calculated.

### Analysis

For each comparison of injury versus untreated controls, we determined the normalised mean difference by calculating the percentage of damage in the injury group. For each comparison of an intervention against injured controls, we calculated a normalised effect size (normalised mean difference) as the percentage of improvement (“+” sign) or worsening (“−” sign) of outcomes [17]. Standard error was calculated as previously described [18].

DerSimonian and Laired random effects weighted mean difference meta-analysis was then used to calculate a summary estimate of injury/improvement magnitude in each and all injury models under various circumstances. Results were presented as the percentage injury against untreated and improvement/worsening of outcome after interventions in injured cohorts with the 95% confidence interval (CI). The variability of the outcomes assessed is presented as the heterogeneity statistic (*Q*) with *n* − 1 degrees of freedom.

The effects of covariates explaining the heterogeneity between studies were assessed using univariate meta-regression with the *metareg* function in STATA/SE10, with a significance level set at *p* < 0.05.

### Study Quality

There were very few study quality checklists designed for in vitro studies. The CRIS Guidelines (Checklist for Reporting In-vitro Studies) were a series of concept notes adapted from the CONSORT guidelines to improve study quality in the dental research [19]. However, these were not sufficient for our purpose. Therefore, we adapted the reviewing criteria designed for the Nature Publication Quality Improvement Project (NPQIP) study [20].

Eight categories were recorded: exclusion, randomisation, blinding, sample size, figures and statistical representation of data, definitions of statistical methods, implementation of statistical methods and measures, as well as reagents and cell preparations. Each category included one or more specific criteria (Supplementary Table 1), and if the publication met the criteria, then 1 point was scored for each item, with a total possible score of 20.

## Results

### Characteristics of Included Studies

**Fig. 1:**
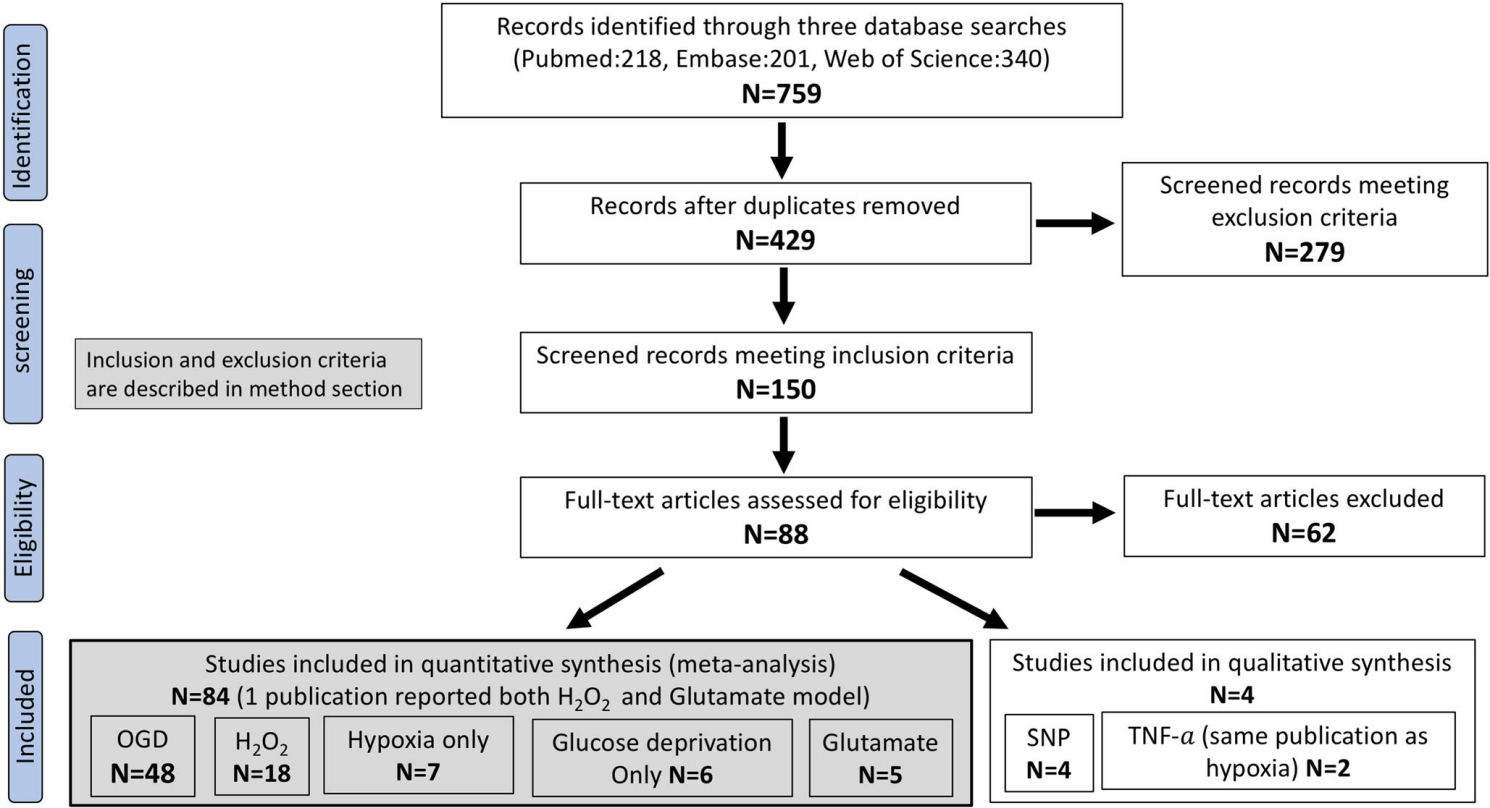
Flow diagram of publication selection. *N* number of publications

Electronic searching identified 759 full publications with 429 potentially relevant articles screened for inclusion after removal of duplicates. After screening, 150 met our prespecified criteria, and 88 were eligible to be included in the analysis. Seven ischaemia-related injury models were described in the included studies. Of these, five models (OGD, H_2_O_2_, hypoxia alone, glucose deprivation alone, glutamate) from 84 publications were included in the meta-analysis (Fig. 1).

OGD was the most commonly used injury model (48 publications, 57.14%), followed by H_2_O_2_ (18 publications, 21.43%), while the other three models made up the remaining 21.43% of the publications (hypoxia only, 7 publications; glucose deprivation only, 6 publications; and glutamate excitotoxicity, 5 publications). One publication reported utilizing both H_2_O_2_ and glutamate-induced injury [21]. Six additional publications were included in a qualitative analysis. Four of these publications used SNP to create oxidative injury and another two used TNF-α in combination with hypoxic injury in the same paper [22, 23]. In the publications providing data for more than one injury model, the publication was counted once and assigned to the injury model group which supplied the most data. However, the individual comparisons were extracted and analysed independently.

### Meta-Analysis of the Effects of Ischaemic Injury Induction Compared with Uninjured Controls

84 publications exploring five ischaemia-related injuries reported cell damage/survival compared with that in uninjured controls in 133 individual comparisons. Injury magnitude was grouped according to the models the authors reported and ranked according to their effect size.

**Fig. 2:**
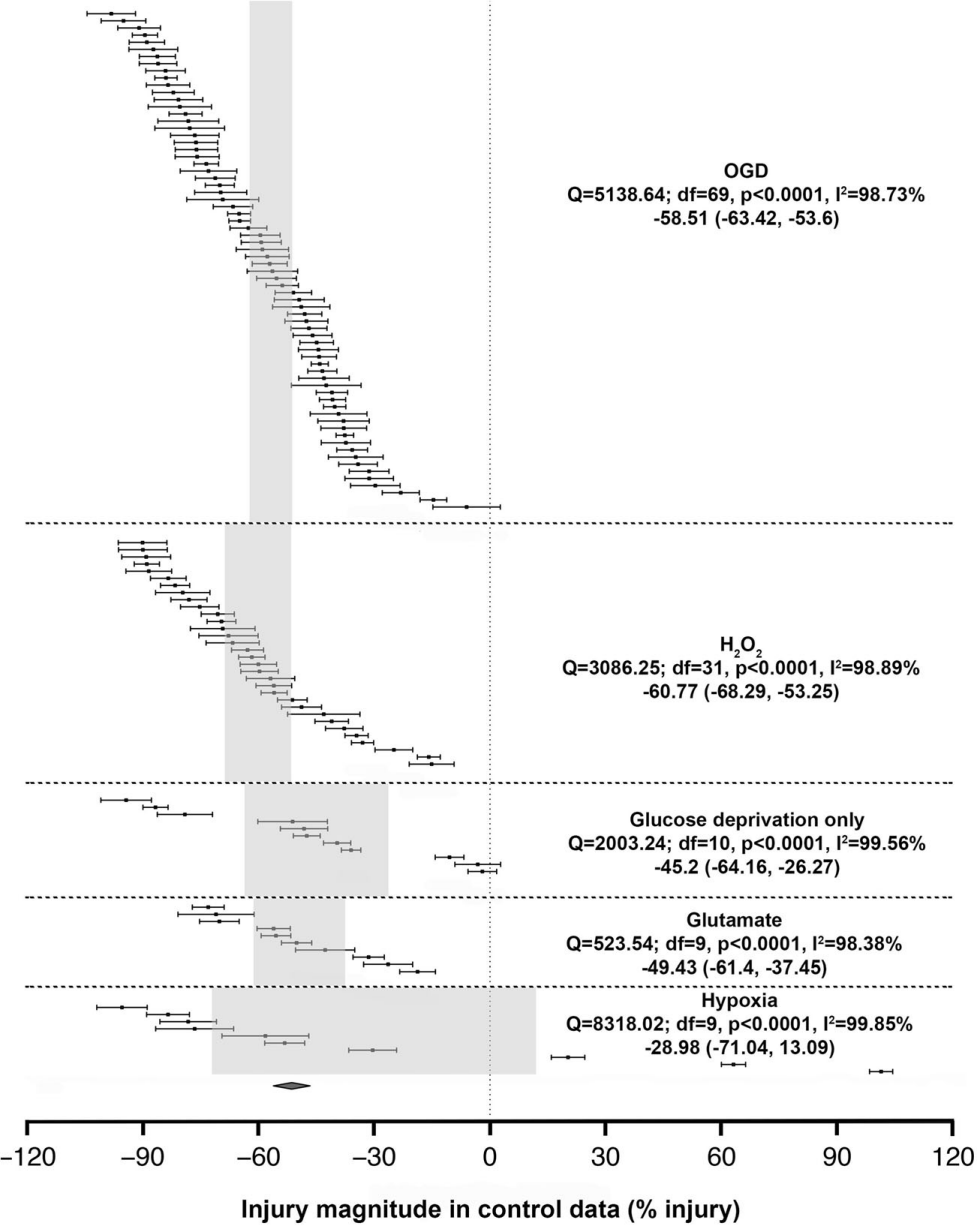
Summary of data included in the meta-analysis with individual comparisons grouped by five in vitro ischaemic models. Data are ranked according to their injury magnitude against untreated controls. The shaded grey bar represents the 95% CI of the individual injuries. The horizontal error bars represent the 95% CI for the individual estimates

There was significant between-study heterogeneity (*I*^2^ = 99.36%, degrees of freedom (*df*) = 132, *p* < 0.0001). The overall injury caused by the different ischaemic-related models was very similar, with an average injury of − 55.04% (95% CI − 59.96, − 50.12) (Fig. 2).

OGD models contributed to the largest number of studies to the dataset (48 publications, 70 comparisons) and exhibited a full-range injury exploration from almost 0 to 100% injury with a mean injury of − 58.51% (95% CI − 63.42, − 53.6). H_2_O_2_ injury (18 publications, 32 comparisons) was used over a narrower injury range with an apparent plateau at − 90% (three comparisons coming from one study) and a mean of − 60.77% (95% CI − 68.29, − 53.25).

Much less data was available for glucose deprivation alone (6 publications, 11 comparisons, − 45.2%, 95% CI − 64.16, − 26.27), glutamate excitotoxicity (5 publications, 10 comparisons, − 49.43%, 95% CI − 61.4, − 37.45) and hypoxia (7 publications, 10 comparisons, − 28.98%, 95% CI − 71.04, 13.09). The glucose deprivation data showed an unusual clustering, suggesting authors to chose low, medium or high level of glucose deprivation for their experiments. While all the aforementioned models reported only damaging effects, the hypoxia data was unusual in having three experimental comparisons (from one paper [24]) which suggested the presence of a hypoxia preconditioning effect. These three models reported less injury than seen after OGD and H_2_O_2_.

### Study Characteristics Accounting for the Heterogeneity of Injury Magnitude

To investigate the factors that influenced injury efficacy, we used meta-regression to identify facets of the experimental design that contributed to outcome heterogeneity. The three outliers from the hypoxia model data suggested a preconditioning effect were excluded from this meta-regression. Three study characteristics accounted for a significant proportion of the between-study heterogeneity in reporting cell damage.

**Fig. 3:**
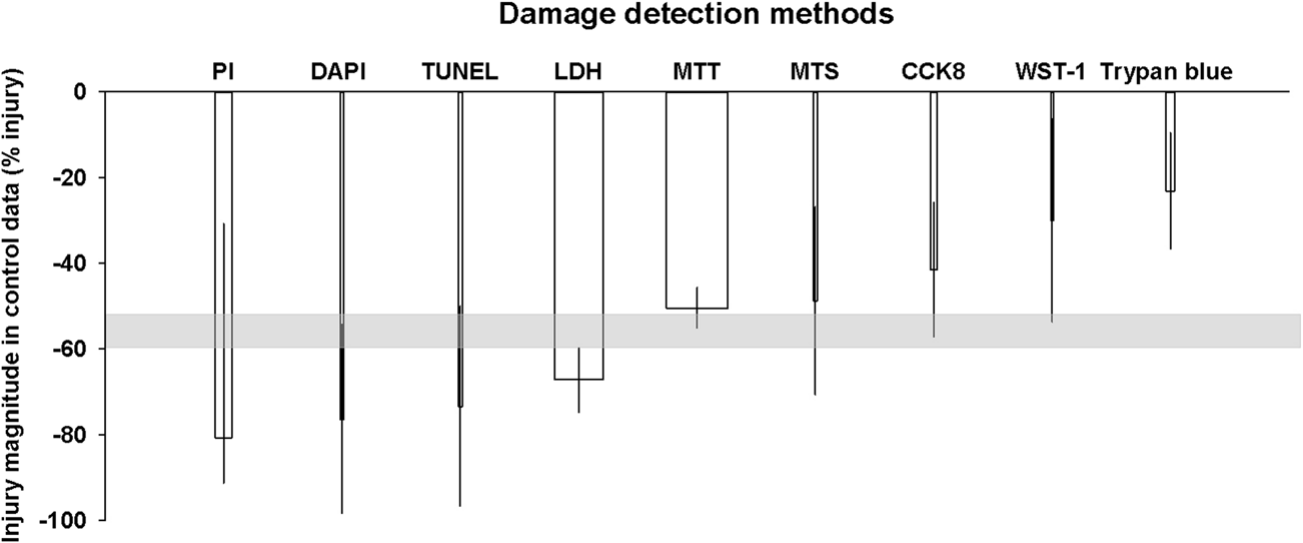
Influence of outcome detection methods on the degree of cellular injury reported in the control cohorts. The shaded grey bar represents the 95% confidence limits of the global estimate. The vertical error bars represent the 95% confidence intervals for the individual estimates. The width of each column represents the volume of available data

The damage detection methodology accounted for most of the observed heterogeneity (*R*^2^ = 44.77%, *p* < 0.000). Nine methods were used to detect cellular injury in the 130 comparisons. The MTT assay was the most frequently used method (52 comparisons, 40% of the data) and detected an average injury of − 50.42% (95% CI − 55.16, − 45.68). By contrast, the second most commonly used method, the LDH assay (41 comparisons, 31.54% of the data), reported − 67.19% injury (95% CI − 74.62, − 59.76). The MTT and LDH assay alone accounted for 71.54% (93 comparisons) of the assessment methods. Overall, methodologies depending on cell counting tend to report more damage (PI − 80.68%, 95% CI − 91.24, − 30.72; DAPI − 76.39%, 95% CI − 98.37, − 54.41). The only exception was the trypan blue exclusion assay (7 comparisons), which appeared to underestimate the injury (− 23.11%, 95% CI − 36.63, − 9.59). Of the other methods, the TUNEL assay reported the highest injury detection (3 comparisons, − 73.52%, 95% CI − 96.63, − 49.94) while the MTS assay (3 comparisons, − 48.75%, 95% CI − 70.75, − 26.74), the CCK8 assay (5 comparisons, − 41.52%, 95% CI − 57.21, − 25.84) and the WST-1 assay (2 comparisons, − 30.02%, 95% CI − 53.66, − 6.38) all reported smaller levels of injury on average (Fig. 3).

**Fig. 4:**
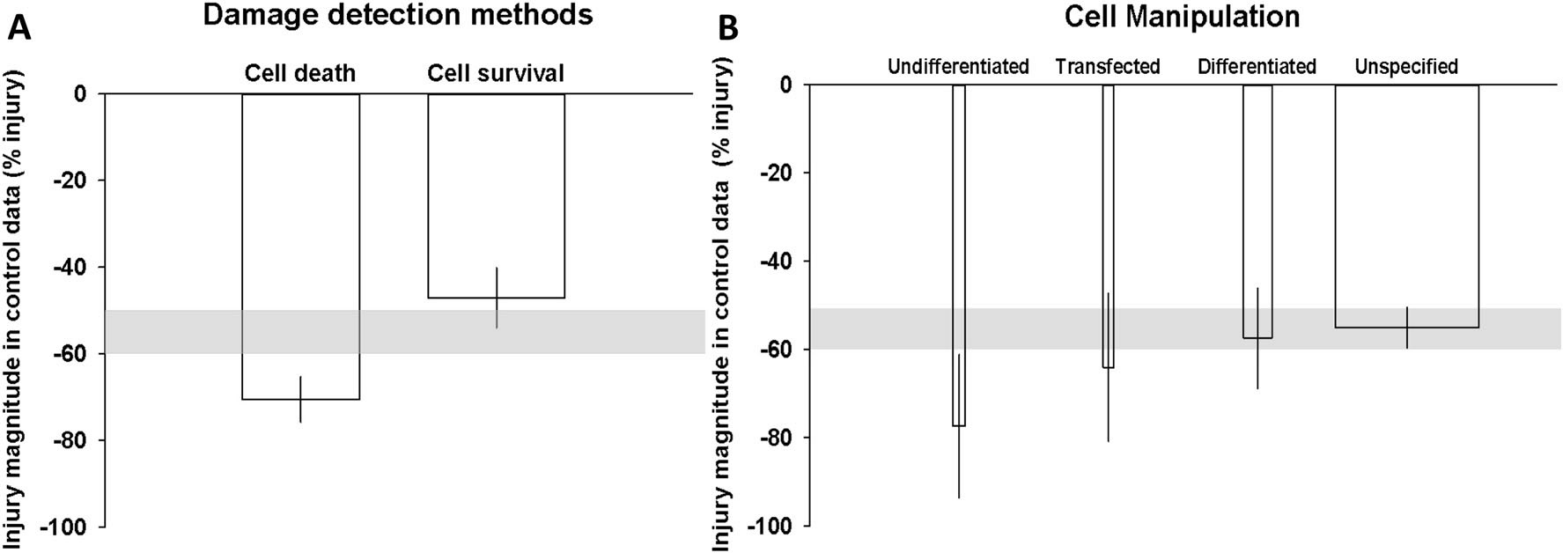
Influence of **a** damage detection methods and **b** prior cell manipulation, on cellular injury detected in untreated control cells. The shaded grey bar represents the 95% confidence limits of the global estimate. The vertical error bars represent the 95% confidence intervals for the individual estimates. The width of each column represents to volume of available data

Each of the nine detection methods could be classified as either measures of cell death (60 comparisons, 46.15% of the data: LDH, DAPI counting, PI (counting and FACS) and TUNEL assay) or survival (70 comparisons, 53.85% of the data: MTT, CCK8, MTS, WST-1 and PI+FDA). Methods belonging to the cell death category tended to report greater injury (− 70.57%, 95% CI − 75.8, − 65.33) than cell survival methods (− 47.14%, 95% CI − 54.07, − 40.2) (*R*^2^ = 28.64%, *p* < 0.000) (Fig. 4a). Of comparisons, 41.67% used multiple detection methods; however, no significant difference in outcome was detected compared to those only assessed by a single method.

The third parameter contributing significantly to the heterogeneity was whether the cells were manipulated prior to experimentation. Such manipulation was common where experiments tried to modify the phenotype of the cells, for example, see Boltze and colleagues [25]. Most publications (96 publications, 73.85%) provided no description of the cell differentiation status. Thirty-four publications (26.15%) reported specifically whether the SH-SY5Y cells were differentiated (19 comparisons), undifferentiated (8 comparisons) or transfected (19 comparisons) (*R*^2^ = 4.13%, *p* < 0.047). Undifferentiated cells were susceptible to the greatest injury (− 77.42%, 95% CI − 93.64, − 61.2) followed by transfected cells (− 64.08%, 95% CI − 80.88, − 47.29). Differentiated cells were less sensitive to injury (− 57.47%, 95% CI − 68.81, − 46.13). Studies where the differentiation status was unspecified showed similar levels of injury to this latter group (− 55.07%, 95% CI − 59.6, − 50.53) (Fig. 4b).

There was no detectable effect of injury duration, reperfusion duration, time of assessment, seeding density, O_2_ concentration used during the injury model or of culture medium used.

### Analysis of OGD and H_2_O_2_ Data Subsets

A large proportion of the data were obtained from just the OGD (48 publications, 70 comparisons) and H_2_O_2_ datasets (18 publications, 32 comparisons). Subgroup analysis revealed that overall heterogeneity (*Q*) fell in these data subsets (total: *Q* = 28,006.95; OGD: *Q* = 5138.64; H_2_O_2_: *Q* = 3086.25).

For the OGD dataset, the damage detection method used accounted for a large part of this heterogeneity (*R*^2^ = 49.87%, *p* = 0.0000). This was consistent with a similar observation from the total dataset. In the H_2_O_2_ data, the type of cell manipulation contributed significantly more to the observed heterogeneity (*R*^2^ = 18.53%, *p* < 0.032, vs total dataset: *R*^2^ = 4.13, *p* < 0.047).

**Fig. 5:**
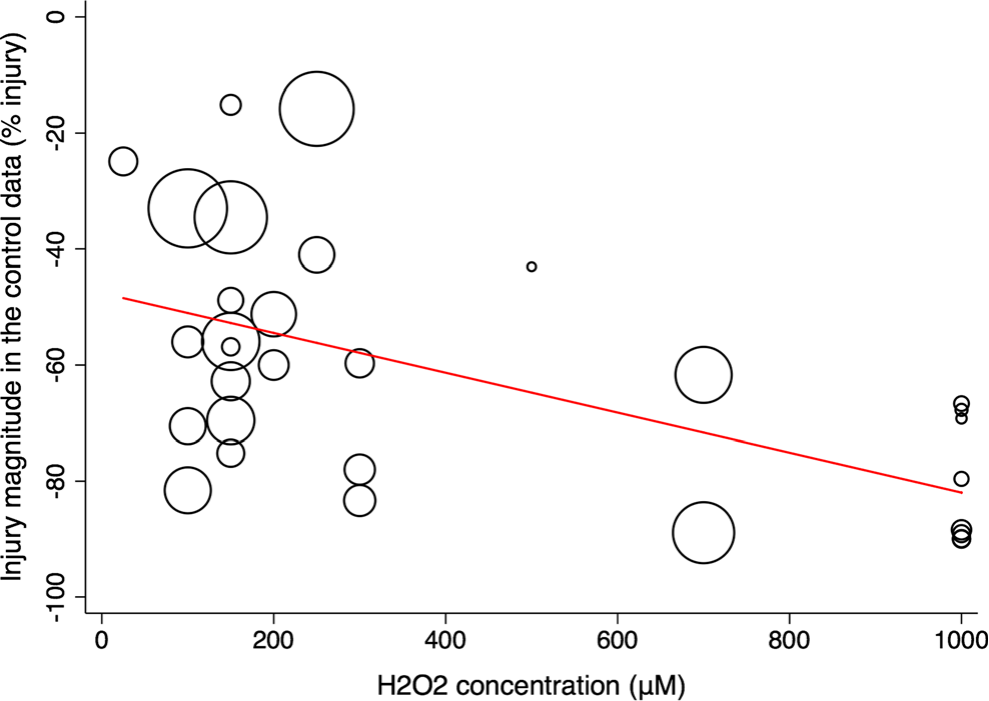
Influence of H_2_O_2_ concentration on injury magnitude in untreated control cohorts. Circle diameter is proportional to study size

For H_2_O_2_-mediated injury, a dose-response relationship contributed significantly to the observed heterogeneity (*R*^2^ = 29.29%, *p* < 0.002). The majority of experiments chose H_2_O_2_ concentrations of less than 200 μM (13 comparisons) and produced a milder injury (− 52.98%, 95% CI − 63.98, − 41.98) than those using concentrations of between 200 and 400 μM (7 comparisons, − 55.07%, 95% CI − 73.57, − 36.58) or 600–800 μM (2 comparisons, − 75.33%, 95% CI − 104.65, − 46.01) and 800–1000 μM (8 comparisons, − 81.18%, 95% CI − 100.69, − 61.66) (Fig. 5). No dose response could be detected for the other injury models.

Other experimental variables (OGD: cell manipulation, gas composition, medium ingredients, injury duration or reperfusion status; H_2_O_2_: damage detection method, injury time or seeding density) did not account for significant proportions of the observed heterogeneity.

### Meta-Analysis of Intervention After In Vitro Ischaemic Injury

In addition to the analysis of experimental variables on the impact on outcomes in untreated controls, we also sought to investigate the effects of the intervention arms of the included studies on outcome.

Eighty publications examined 169 intervention comparisons from the top five in vitro ischaemia models. Four publications (one using glucose deprivation [26], one using hypoxia [25], and two using OGD [27, 28]) were excluded because no intervention was reported or it was reported in non-ischaemic models. The OGD model was used to study the largest number of interventions (88 comparisons), followed by H_2_O_2_ (33 comparisons), hypoxia (15 comparisons), glutamate excitotoxicity (10 comparisons) and glucose deprivation alone (7 comparisons). Multiple interventions were frequently reported in one publication, and they were treated as individual comparisons for the purposes of this analysis (Supplementary Table 6).

**Fig. 6:**
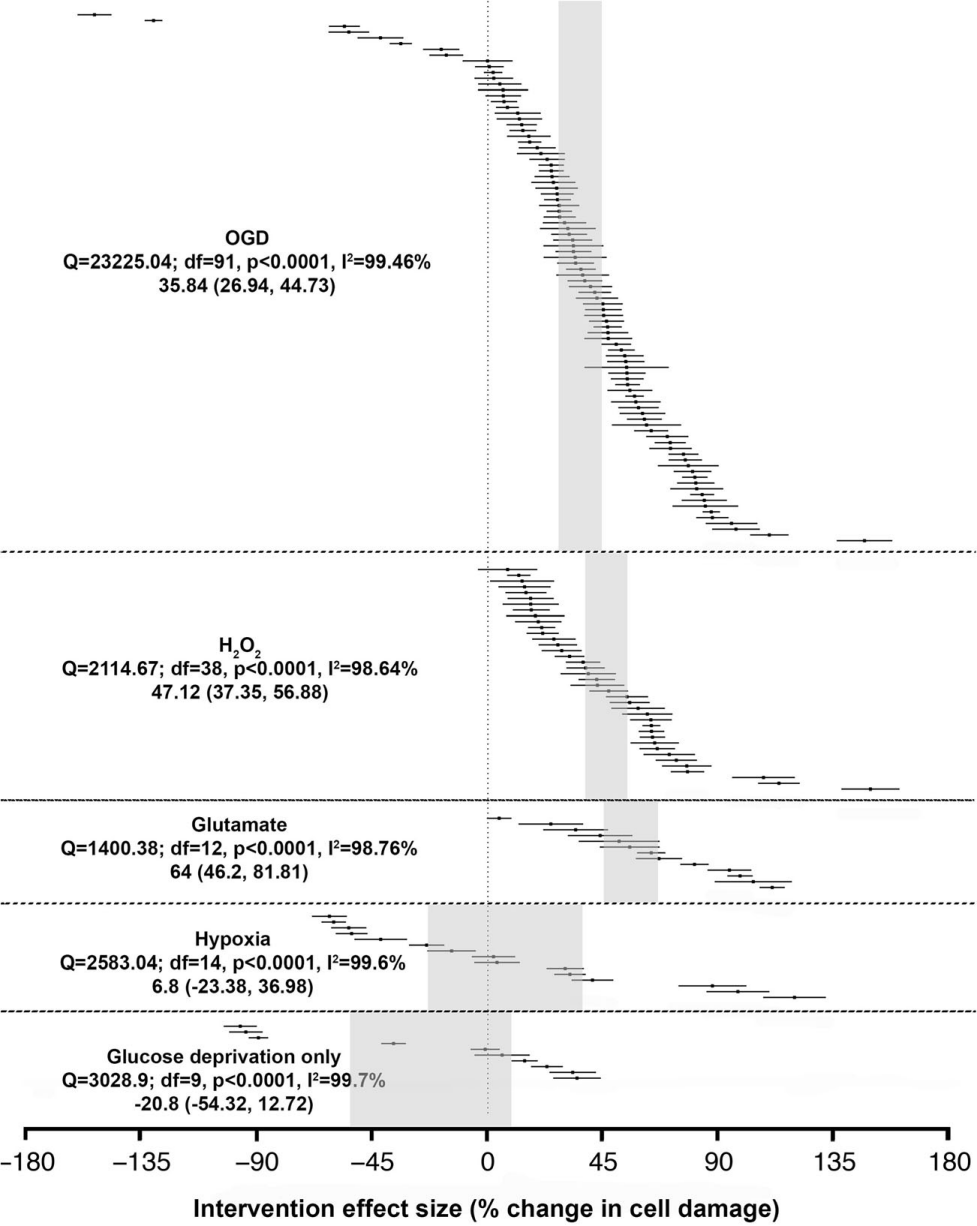
Summary of interventional data included in the meta-analysis from five in vitro ischaemic models. Data are ranked according to their effect on change after cell damage from each injury. The shaded grey bar represents the 95% CI of the individual injuries. The vertical error bars represent the 95% CI for the individual estimates

Heterogeneity was greater overall where an intervention was applied (*Q* = 42,108, 169 comparisons) than for the data for injury alone in untreated cohorts (*Q* = 28,006 over 133 comparisons). Overall, the interventions improved cell survival by 34.67% (95% CI 27.7, 41.62, *I*^2^ = 99.48%, *p* < 0.0001) across the five models. Interestingly, glutamate and H_2_O_2_ injury were the only two models where only improved outcomes were reported with 64% (95% CI 46.2, 81.81) and 47.12% (95% CI 37.35, 56.88) improvement, respectively. In contrast, negative outcomes were common in the models of OGD and its subcomponent models (hypoxia and glucose deprivation only), and thus led to smaller effect sizes (OGD 35.84%, 95% CI 26.94, 44.73; hypoxia 6.8%, 95% CI − 23.38, 36.98). Glucose deprivation was the only model to give rise an overall worsening of outcome after the interventions (− 20.8%, 95% CI − 54.32, 12.72) (Fig. 6).

Overall, we found that 21/169 comparisons reported negative outcomes of which 10 involved genome manipulations. These studies appeared to be consistent with the authors who intent to study mechanisms.

Of all intervention comparisons, 24.85% utilized herbal or traditional medicines or their derivates which improved outcome by 65.12% on average. Of the studies reporting the greatest improvement (> 90% effect size), more than half (7/13 comparisons) were studies of the bioactive constituents of herbal medicines (Supplementary Table 2).

### Mechanism of Action of Interventions Studied

The most commonly studied molecules were only represented in two or three independent studies. Therefore, there was insufficient data for meta-analytical exploration of their comparative effects.

When we grouped intervention targets into single or mixed ischaemia-related mechanisms according to the author’s original statements, we found that 91/169 (53.85%) experiments studied interventions targeting a single mechanism, of which half (46/91 comparisons, 50.55%) targeted apoptosis. This was followed by antioxidant-related mechanisms (32/91 comparisons, 35.16%), which was also the only targeting mechanism studied in all the aforementioned models. The remainder (14%) studied excitotoxicity: 3 comparisons, mitochondrial protection; 3 comparisons, growth factor secretion; 4 comparisons; and axonal growth, 3 comparisons (Supplementary Table 3).

Interventions targeting multiple mechanisms were investigated in 78 comparisons. Seventy-six comparisons of these studied two mechanisms in 11 different combinations. One study referred to three potential mechanisms for their interventional drugs but no experiments to identify the mechanism were performed [9]. Drugs modifying apoptosis and oxidative biology were the most commonly studied pairs (32/76 comparisons, 42.11%). This was particularly true for the H_2_O_2_ injury studies where 17/17 studies all used such a combination (Supplementary Table 4).

Researchers using the OGD injury model studied the greatest range of intervention mechanisms in combination (9/11 mechanisms). Apoptosis was the most commonly studied mechanism both singly (40/52 comparisons, 77%) or in combination with antioxidant interventions (both have 13 comparisons, 32.5%). This pattern was reversed for H_2_O_2_ injury (14/22 comparisons, 63.64%, studied antioxidant mechanisms singly or 17/17 comparisons, 100%, studied antioxidant and anti-apoptotic mechanisms).

### Study Quality

In systematic review and meta-analysis, which is most often used to determine a therapeutic effect in clinical studies, a key determinant of the value of the analysis is the quality of the included studies. This is most often ascertained by formal risk of bias analysis [29] or by application of a simple checklist in pre-clinical studies, where understanding of the need to report such issues lags behind the clinical field. However, in in vitro research, these issues have not gained widespread recognition as potential sources of experimental error. Therefore, in our study, we had only a limited ability to assess study quality.

We reported and scored the items using our modified study quality checklist for each included publication from the five most commonly used ischaemia-related models (Supplementary Table 5). Study design features which help reduce bias, such as randomisation, blinding, sample size calculation and description of inclusion and exclusion criteria, were extremely poorly reported. Only one publication in H_2_O_2_ injury and one publication on OGD injury out of a total of 84 publications reported their exclusion criteria (2.38%), and the same study was also the only study to report a sample size calculation (1.19%). None of the publications reported either randomisation or blinding in their full text. While “*N*” was reported in more than 95% of the publications, there was generally a poor description of what “*N*” stood for (wells/plates/microscope fields). Only 19/84 publications (22.62%) gave a clear statement of whether the samples represented technical or biologically relevant replicates, and 29/84 (34.52%) described how many times the experiment was replicated. Cell preparation was also poorly reported, with only half of the publications (54.76%) describing the sources of their cell lines. Mycoplasma contamination testing and cell line authentication were not reported in any of the included publications.

Using the modified NPQIP score sheet (maximum score 20) [20], the mean quality score for each of the models did not differ significantly, with glucose deprivation achieving the highest score of 8.67 followed by three similar models: H_2_O_2_, 7.56; OGD, 7.33; and glutamate, 7.4. The hypoxia model gave rise the lowest score, at only 6.86.

In the future, in vitro researchers need to determine whether such issues impact the validity of their results.

## Discussion

Initial scoping of the citation databases indicated that our desire to use systematic review and meta-analysis to explore and ultimately understand the in vitro data available on the human ischaemic cascade was likely to prove intractable because of the number and heterogeneity of the available publications.

Therefore, we used a word frequency analysis of the titles and abstracts of these published data to refine our question. This analysis revealed that the SH-SY5Y cell line was the most commonly used tool in human in vitro ischaemic research.

Systematic review and meta-analysis of the studies using this cell line indicated that the use of five models of ischaemia-related injury dominated this literature: oxygen and glucose deprivation, H_2_O_2_-induced oxidative stress, oxygen deprivation, glucose deprivation and glutamate excitotoxicity.

The pattern of outcomes revealed important information about both researchers’ experimental choices and the biology of the systems. In the control arms of the publications, there was substantial heterogeneity. Nearly half of this could be explained by the methods used to determine cell fate with methods detecting cell death producing greater estimates of damage than methods detecting the proportion of survival cells. We also found that damage detection methods utilizing microscopic subfield analysis tended to detect more injury compared with “whole-well”-based analysis with the exception of the trypan blue method.

Whether the cells had been manipulated prior to the injury also accounted for a significant proportion of heterogeneity with undifferentiated cells appearing to be more susceptible to injury than differentiated neurons. For H_2_O_2_-injured cells, the type of cell manipulation accounted for nearly 20% of the observed heterogeneity. For the majority of publications, there was no clear description of the differentiation status of the cells used and hence, we classified this as “unspecified”. In the overall analysis, this large group behaved very differently from the cells specified as undifferentiated. However, of the eight studies using undifferentiated cells, the worst outcomes were seen in those without the equivalent of a reperfusion period; this suggests the possibility that oxygenation status might offer an explanation. However, further work is required to clarify this issue. Certainly, we would not expect normal human neural progenitors to be more sensitive to injury than their highly arborised and electrically active mature derivatives as observation found that differentiated cells require more oxygen and are therefore more susceptible to injury [30].

For H_2_O_2_-injured cells, a clear dose-response relationship was detectable. Such a response was not evident for the other injuries and nor was there a clear influence of injury duration. For OGD and the related hypoxia and glucose deprivation models, this might be interpreted as the result of the use of a highly optimized set of uniform experimental conditions. However, the broad range of levels of damage detected in these control circumstances (from nearly 0 to 100% injury) does not support this interpretation. Indeed, for hypoxia alone, the data suggested that under some circumstances, a protective effect is possible. The highly clustered range of outcomes for just glucose deprivation also suggests some unidentified systematic effect.

Interestingly, for both H_2_O_2_- and glutamate-induced injury, there appeared to be a ceiling effect, suggesting that a proportion of the cultured cells were resistant to these injuries.

SH-SY5Y is a highly proliferate cell line, and others have previously reported that seeding density influences outcomes [31]. However, we found no relationship between starting seeding density (53.6% of studies reported this data) and the outcomes.

Analysis of the intervention arms of the included publications proved more difficult than expected because of the breadth of interventions studied (Supplementary Table 6). No single “drug” was represented sufficiently in the dataset to estimate the effect size for comparison purposes. Grouping interventions by the broad mechanistic classes indicated in the originating authors’ text did however provide useful data. There was an overwhelming focus on apoptotic and oxidative biology mechanisms. Given the range of mechanisms targeted in preclinical animal studies [1] and in clinical trials [32] for ischaemic stroke, this was surprising. Also surprising was the high proportion of studies on herbal or traditional medicines and their high ranking in terms of protective effect. Overall, the lack of specificity by authors on whether their study was to explore a molecule therapeutic potential or whether the experimental purpose was to explore mechanisms of injury made in-depth analysis of this impractical.

In clinical and preclinical animal systematic review and meta-analysis, assessment of the risk of bias within a publication or cohort of publications provides important data allowing readers to judge whether the work is worthy of further investigation (follow-up experiments, progress to clinical trials). In this cohort of publications, reporting of these issues (randomisation, blinding, inclusion and exclusion criteria, a priori power calculations) is so poor that we have to conclude that the risk of bias is very high. A recent systematic review of the use of SH-SY5Y to model Parkinson’s disease did not perform meta-analysis and made no comments of the likely quality of the studies included in the review [33].

## Electronic supplementary material

Supplementary Table 1 (DOCX 14 kb)

Supplementary Table 2 (DOCX 15 kb)

Supplementary Table 3 (DOCX 13 kb)

Supplementary Table 4 (DOCX 13 kb)

Supplementary Table 5 (DOCX 16 kb)

Supplementary Table 6 (XLSX 27 kb)

Appendix 1 (DOCX 13 kb)

Appendix 2 (DOCX 14 kb)
