# Supplementary material for: Human Ischaemic Cascade Studies Using SH-SY5Y Cells: a Systematic Review and Meta-Analysis
Source: Transl Stroke Res. 2018 Mar 23;9(6):564–74. doi: 10.1007/s12975-018-0620-4 (PMC6208743; doi:10.1007/s12975-018-0620-4)
Supplement: Supplementary file 1 — (DOCX 14 kb) [file 12975_2018_620_MOESM1_ESM.docx]

| **Assessment categories** | **Scoring details** | **Score if meets corresponding criteria** |
| --- | --- | --- |
| **Exclusions** | A description of if samples were excluded from the analysis | 1 |
| **Randomization** | A description of which method of randomization was used to determine how samples were allocated to experimental groups | 1 |
| **Blinding** | A description of whether the investigator was blinded to the group allocation during the experiment and/or when assessing the outcome | 1 |
| **Sample Size** | A description of how the sample size was chosen to ensure adequate power to detect a pre-specified effect size | 1 |
| **Figures and statistical representation of data** | The exact sample size (n) for each experimental group/condition was given as a number, not a range | 1 |
|  | A description of whether the samples represent technical or biological replicates | 1 |
|  | A statement of how many times the experiment shown was replicated | 1 |
| **Definitions of statistical methods and measures** | The summary estimates are defined as a median or average | 1 |
|  | The error bars are defined as s.d., s.e.m. or c.i. | 1 |
|  | Common test (such as t-test, simple χ2 tests, Wilcoxon and Mann-Whitney tests, and any form of ANOVA testing), if not a common test, the test is described in the methods section | 1 |
|  | If the statistical test used is a t or z test, this was reported as one sided or two sided | 1 |
|  | Adjustments for multiple comparisons are applied where this is appropriate | 1 |
|  | The statistical test results (e.g., P values, F statistic etc.) are presented | 1 |
| **Implementation of statistical methods and measures** | The authors show that their data meet the assumptions of the tests | 1 |
|  | An estimate of variation is reported within each group of data | 1 |
|  | The variance is similar (difference less than two-fold) between the groups that are being statistically compared | 1 |
| **Reagents and cells** | Every antibody used in the manuscript been profiled for use in the system under study by either citation, catalog number, clone number or validation profile | 1 |
|  | The source of cell lines was provided | 1 |
|  | The authors report whether the lines used have been authenticated recently | 1 |
|  | The authors report whether the lines used have been tested for mycoplasma contamination recently (within 6 months of use) | 1 |
| Total |  | 20 |

**Supplementary table 1: Study quality checklist (refer to the criteria for in vitro study in NPQIP)**
