# Supplementary material for: Human Ischaemic Cascade Studies Using SH-SY5Y Cells: a Systematic Review and Meta-Analysis
Source: Transl Stroke Res. 2018 Mar 23;9(6):564–74. doi: 10.1007/s12975-018-0620-4 (PMC6208743; doi:10.1007/s12975-018-0620-4)
Supplement: Supplementary file 2 — (DOCX 15 kb) [file 12975_2018_620_MOESM2_ESM.docx]

| **Intervention** | **Mechanism categories** | **Effect size** | **SEM** | **Publication** |
| --- | --- | --- | --- | --- |
| Ginsenoside Rg1 | Herbal medicine (Ginsenoside) | 149.06 | 32.08 | Protective effects of ginsenoside Rg1 against hydrogen peroxide-induced injury in human neuroblastoma cells |
| Metabolite monomethyl fumarate (MMF) | Fumaric acid esters | 146.75 | 29.21 | Fumaric acid esters promote neuronal survival upon ischemic stress through activation of the Nrf2 but not HIF-1 signaling pathway |
| siRNA Thioredoxin 1 (Trx1) | mediators of cell functions via regulation of the thiol redox state | 119.48 | 37.31 | Thioredoxin 1 and glutaredoxin 2 contribute to maintain the phenotype and integrity of neurons following perinatal asphyxia |
| Danshen derivates 8 | Herbal medicine (Danshen) | 113.41 | 16.24 | Neuroprotective effect of Danshensu derivatives as anti-ischaemia agents on SH-SY5Y cells and rat brain |
| Kukoamine B | Herbal medicine (bioactive component of cortex lycii radicis) | 110.78 | 5.72 | Kukoamine B, an amide alkaloid, protects against NMDA-induced neurotoxicity and potential mechanisms in vitro |
| Cordyceps sinensis Oral Liquid (CSOL) | Herbal medicine (Cordyceps sinensis Oral Liquid, CSOL) | 109.68 | 13.73 | Cordyceps sinensis Oral Liquid Inhibits Damage Induced by Oxygen and Glucose Deprivation in SH-SY5Y Cells |
| DJ-1knock down and glutathione S-transferase-tagged recombinant human DJ-1 protein (GST-DJ-1) | Oxidative states | 107.39 | 37.45 | Oxidative stress induction of DJ-1 protein in reactive astrocytes scavenges free radicals and reduces cell injury |
| mouse adipose-derived stem cells- conditioned medium (mASC-CM) | conditioned medium (CM) | 103.41 | 56.98 | The conditioned medium of murine and human adipose-derived stem cells exerts neuroprotective effects against experimental stroke model |
| Kukoamine B | Herbal medicine (bioactive component of cortex lycii radicis) | 98.25 | 5.8 | Kukoamine B, an amide alkaloid, protects against NMDA-induced neurotoxicity and potential mechanisms in vitro |
| Adrenomedullin (AM)+Adrenomedullin binding protein (AMBP-1) | AM: vasodilator peptide hormone | 97.43 | 37.76 | Circulating hormone adrenomedullin and its binding protein protect neural cells from hypoxia-induced apoptosis |
| Baicalin | Herbal medicine (Baicalin) | 96.67 | 21.34 | Pretreatment with baicalin attenuates hypoxia and glucose deprivation-induced injury in SH-SY5Y cells |
| Monocyte locomotion inhibitory factor (MLIF) | a heat-stable pentapeptide | 94.93 | 25.18 | MLIF alleviates SH-SY5Y neuroblastoma injury induced by oxygen-glucose deprivation by targeting eukaryotic translation elongation factor 1A2 |
| MLIF alleviates SH-SY5Y neuroblastoma injury induced by oxygen-glucose deprivation by targeting eukaryotic translation elongation factor 1A2 | Herbal medicine (chuanxiong's component: Tetramethylpyrazine (TMP)) | 94.12 | 18.03 | A Tetramethylpyrazine Piperazine Derivate CXC137 Prevents Cell Injury in SH-SY5Y Cells and Improves Memory Dysfunction of Rats with Vascular Dementia |

**Supplementary table 2: Summary of the interventions reporting the greatest improvement (>90% effect size).**
