# Supplementary material for: Human Ischaemic Cascade Studies Using SH-SY5Y Cells: a Systematic Review and Meta-Analysis
Source: Transl Stroke Res. 2018 Mar 23;9(6):564–74. doi: 10.1007/s12975-018-0620-4 (PMC6208743; doi:10.1007/s12975-018-0620-4)
Supplement: Supplementary file 3 — (DOCX 13 kb) [file 12975_2018_620_MOESM3_ESM.docx]

| **Mechanisms (numbers)** | **Mechanism**  **subgroup** | **Numbers** | **OGD** | **H_2_O_2_** | **Hypoxia** | **Glutamate** | **Glucose deprivation** |
| --- | --- | --- | --- | --- | --- | --- | --- |
| Excitotoxicity  (3) | NMDAR antagonist | 1 |  |  |  | 1 |  |
|  | Glutamate toxicity | 2 |  |  |  | 2 |  |
| Oxidative stress (35) | Mitochondrial protection | 3 |  | 1 | 2 |  |  |
|  | Antioxidant | 32 | 9 | 14 | 8 | 1 | 0 |
| Cell death (46) | Via apoptosis | 46 | 40 | 4 | 2 |  | 0 |
| Secreting growth factor (4) | - | 4 |  | 3 |  | 1 |  |
| Axonal growth (3) | - | 3 | 3 (1 publication) |  |  |  |  |
| Total |  | 91 | 52 | 22 | 12 | 5 | 0 |

**Supplementary table 3. Summary of intervention mechanisms studied singly in each injury model.**
