# Supplementary material for: Human Ischaemic Cascade Studies Using SH-SY5Y Cells: a Systematic Review and Meta-Analysis
Source: Transl Stroke Res. 2018 Mar 23;9(6):564–74. doi: 10.1007/s12975-018-0620-4 (PMC6208743; doi:10.1007/s12975-018-0620-4)
Supplement: Supplementary file 4 — (DOCX 13 kb) [file 12975_2018_620_MOESM4_ESM.docx]

| **Mechanisms (numbers)** | **Mechanism**  **subgroup** | **Numbers** | **OGD** | **H_2_O_2_** | **Hypoxia** | **Glutamate** | **Glucose deprivation** |
| --- | --- | --- | --- | --- | --- | --- | --- |
| Excitotoxicity  (13) | NMDAR antagonist+ oxidative stress | 4 |  |  |  | 4 |  |
|  | NMDAR antagonist+  cell death | 5 | 3 |  |  | 2 |  |
|  | excitotoxicity+  cell death | 4 | 4 |  |  |  |  |
| Oxidative stress (44) | ER stress+ oxidative stress | 6 | 4 |  |  |  | 2 |
|  | ER stress+ cell death | 3 | 1 |  |  |  | 2 |
|  | ER stress+ autophagy | 1 |  |  |  |  | 1 |
|  | Oxidative stress+  cell death | 32 | 13 | 17 |  | 2 |  |
|  | Autophagy+ cell death | 2 | 2 |  |  |  |  |
| Inflammation (14) | Inflammation+  oxidative stress | 2 | 2 |  |  |  |  |
|  | Inflammation+ cell death | 12 | 6 |  | 3 |  | 3 |
| Cell death (5) | Cell death via apoptosis+ other type of cell death | 5 | 5 |  |  |  | 0 |
| Total |  | 76 | 40 | 17 | 3 | 8 | 8 |

**Supplementary table 4. Summary of intervention mechanisms studied in combination in each injury model.**
