# Supplementary material for: Human Ischaemic Cascade Studies Using SH-SY5Y Cells: a Systematic Review and Meta-Analysis
Source: Transl Stroke Res. 2018 Mar 23;9(6):564–74. doi: 10.1007/s12975-018-0620-4 (PMC6208743; doi:10.1007/s12975-018-0620-4)
Supplement: Supplementary file 5 — (DOCX 16 kb) [file 12975_2018_620_MOESM5_ESM.docx]

| **Assessment categories** | **Scoring details** | Glucose deprivation (6) | Glutamate  (5) | H_2_O_2_  (18) | Hypoxia  (7) | OGD  (48) | Total Numbers (% in total) |
| --- | --- | --- | --- | --- | --- | --- | --- |
| **Exclusions** | A description of if samples were excluded from the analysis | 0 | 0 | 1  5.56% | 0 | 1  2.08% | 2  2.38% |
| **Randomization** | A description of which method of randomization was used to determine how samples were allocated to experimental groups | 0 | 0 | 0 | 0 | 0 | 0 |
| **Blinding** | A description of whether the investigator was blinded to the group allocation during the experiment and/or when assessing the outcome | 0 | 0 | 0 | 0 | 0 | 0 |
| **Sample Size** | A description of how the sample size was chosen to ensure adequate power to detect a pre-specified effect size | 0 | 0 | 0 | 0 | 1  2.08% | 1  1.19% |
| **Figures and statistical representation of data** | The exact sample size (n) for each experimental group/condition was given as a number, not a range | 6  100% | 5  100% | 18  100% | 7  100% | 46 95.83% | 82 97.62% |
|  | A description of whether the samples represent technical or biological replicates | 2  33.3% | 1  20% | 7  38.89% | 4  57.14% | 5  10.42% | 19 22.62% |
|  | A statement of how many times the experiment shown was replicated | 1  16.67% | 2  40% | 8  44.44% | 3  42.86% | 15 31.25% | 29 34.52% |
| **Definitions of statistical methods and measures** | The summary estimates are defined as a median or average | 6  100% | 5  100% | 18  100% | 7  100% | 48  100% | 84  100% |
|  | The error bars are defined as s.d., s.e.m. or c.i. | 6  100% | 5  100% | 18  100% | 7  100% | 29 60.42% | 65 77.38% |
|  | Common test (such as t-test, simple χ2 tests, Wilcoxon and Mann-Whitney tests, and any form of ANOVA testing), if not a common test, the test is described in the methods section | 6  100% | 5  100% | 15  83.33% | 5  71.43% | 44 91.76% | 75 89.29% |
|  | If the statistical test used is a t or z test, this was reported as one sided or two sided | 0 | 0 | 1  5.56% | 0 | 5  10.42% | 6  7.14% |
|  | Adjustments for multiple comparisons are applied where this is appropriate | 6  100% | 5  100% | 5  27.78% | 2  28.75% | 32 66.67% | 50 59.52% |
|  | The statistical test results (e.g., P values, F statistic etc.) are presented | 6  100% | 5  100% | 18  100% | 7  100% | 48  100% | 84  100% |
| **Implementation of statistical methods and measures** | The authors show that their data meet the assumptions of the tests | 0 | 0 | 0 | 0 | 2  4.17% | 2  2.38% |
|  | An estimate of variation is reported within each group of data | 0 | 0 | 0 | 0 | 3  6.25% | 3  3.57% |
|  | The variance is similar (difference less than two-fold) between the groups that are being statistically compared | 3  50% | 4  80% | 13  72.22% | 4  57.14% | 35 72.92% | 59 70.24% |
| **Reagents and cells** | Every antibody used in the manuscript been profiled for use in the system under study by either citation, catalog number, clone number or validation profile | 0 | 0 | 5 27.78% | 0 | 7  14.58% | 18 21.43% |
|  | The source of cell lines was provided | 6  100% | 0 | 9  50% | 2  28.57% | 31 64.58% | 46 54.76% |
|  | The authors report whether the lines used have been authenticated recently | 0 | 0 | 0 | 0 | 0 | 0 |
|  | The authors report whether the lines used have been tested for mycoplasma contamination recently (within 6 months of use) | 0 | 0 | 0 | 0 | 0 | 0 |
| **Average score**  (out of total score:20) |  | 8.67 | 7.4 | 7.56 | 6.86 | 7.33 |  |

**Supplementary table 5: Study characteristics accounting for the heterogeneity of injury magnitude in all the ischaemic-related models.**
