# Supplementary material for: Human Ischaemic Cascade Studies Using SH-SY5Y Cells: a Systematic Review and Meta-Analysis
Source: Transl Stroke Res. 2018 Mar 23;9(6):564–74. doi: 10.1007/s12975-018-0620-4 (PMC6208743; doi:10.1007/s12975-018-0620-4)
Supplement: Supplementary file 7 — (DOCX 13 kb) [file 12975_2018_620_MOESM7_ESM.docx]

**Pubmed**

brain ischemia [All Fields] OR brain ischaemia [All Fields] OR brain ischemic [All Fields]OR brain infarctions [All Fields]OR brain infarction [All Fields]OR cerebral infarction [All Fields]OR cerebral infarctions [All Fields]OR stroke [All Fields]OR ischemic stroke [All Fields]OR Brain Ischemia [MeSH Terms]OR Stroke [MeSH Terms]OR Cerebral infarction [MeSH Terms]

AND

Apoptosis [All Fields] OR Necrosis [All Fields] OR Cell death[All Fields] OR caspases[All Fields] OR Enzyme activation[All Fields] OR Apoptosis [MeSH Terms] OR Necrosis[MeSH Terms] OR Cell death[MeSH Terms] OR caspases[MeSH Terms] OR Enzyme activation[MeSH Terms]

OR

Free radicals [All Fields] OR Mitochondria [All Fields] OR oxidative stress [All Fields] OR reactive oxygen species [All Fields] OR Nitric Oxide [All Fields] OR nitric oxide synthase [All Fields] OR lipid peroxidation [All Fields] OR NADPH oxidase [All Fields] OR reactive nitrogen species [All Fields] OR antioxidant [All Fields] OR free radical scavenger [All Fields] OR Free radicals [MeSH Terms] OR Mitochondria [MeSH Terms] OR oxidative stress [MeSH Terms] OR reactive oxygen species [MeSH Terms] OR Nitric Oxide [MeSH Terms] OR nitric oxide synthase [MeSH Terms] OR lipid peroxidation [MeSH Terms] OR NADPH oxidase [MeSH Terms] OR reactive nitrogen species [MeSH Terms] OR antioxidant [MeSH Terms] OR free radical scavenger [MeSH Terms]

OR

Excitatory amino acid [All Fields] OR glutamate acid [All Fields] OR excitatory amino acid transporter [All Fields] OR receptors, neurotransmitter [All Fields] OR receptors, glutamate [All Fields] OR receptors N-Methyl-D-Aspartate [All Fields] OR receptors, AMPA [All Fields] OR Neurons [All Fields] OR astrocytes [All Fields] OR oligodendroglia [All Fields] OR synapses [All Fields] OR Cortical spreading depression [All Fields] OR anoxia[All Fields] ] OR calcium signalling [All Fields] OR Calcium[All Fields] OR Excitatory amino acid [MeSH Terms] OR glutamate acid [MeSH Terms] OR excitatory amino acid transporter [MeSH Terms] OR receptors, neurotransmitter [MeSH Terms] OR receptors, glutamate [MeSH Terms] OR receptors N-Methyl-D-Aspartate [MeSH Terms] OR receptors, AMPA [MeSH Terms] OR Neurons [MeSH Terms] OR astrocytes [MeSH Terms] OR oligodendroglia [MeSH Terms] OR synapses [MeSH Terms] OR Cortical spreading depression [MeSH Terms] OR anoxia[MeSH Terms] OR calcium signalling [MeSH Terms] OR Calcium[MeSH Terms]

OR

Inflammation [All Fields] OR Inflammation Mediators [All Fields] OR Inflammatory [All Fields] OR neurogenic inflammation [All Fields] OR Chemokines [All Fields] OR Cytokines [All Fields] OR RNA [All Fields] OR MicroRNAs [All Fields] OR complement system protein [All Fields] OR Leukocytes [All Fields] OR Neutrophils [All Fields] OR Microglia [All Fields] OR Interlukins [All Fields] OR Inflammation [MeSH Terms] OR Inflammation Mediators [MeSH Terms] OR neurogenic inflammation [MeSH Terms] OR Chemokines [MeSH Terms] OR Cytokines [MeSH Terms] OR RNA [MeSH Terms] OR MicroRNAs [MeSH Terms] OR complement system protein [MeSH Terms] OR Leukocytes [MeSH Terms] OR Neutrophils [MeSH Terms] OR Microglia [MeSH Terms] OR Interlukins[MeSH Terms]

**Embase**

brain ischemia [mp.] OR brain ischaemia [mp.] OR brain ischemic [mp.]OR brain infarctions [mp.]OR brain infarction [mp.] OR cerebral infarction [mp.] OR cerebral infarctions [mp.]OR brain ischemia [mp.] OR cerebrovascular accident [mp.] OR brain ischemia [MeSH Terms] OR cerebrovascular accident [MeSH Terms] OR brain infarction [MeSH Terms]

mp. [mp=title, abstract, subject headings, heading word, drug trade name, original title, device manufacturer, drug manufacturer, device trade name, keyword]

AND

Apoptosis [mp.] OR Necrosis [mp.] OR Cell death[Mp.] OR caspases [mp.] OR Enzyme activation[mp.] OR Apoptosis [MeSH Terms] OR Necrosis[MeSH Terms] OR Cell death[MeSH Terms] OR caspases[MeSH Terms] OR Enzyme activation[MeSH Terms] OR Cortical spreading depression [mp.] OR anoxia[mp.] OR Cortical spreading depression [MeSH Terms] OR anoxia[MeSH Terms]

OR

Free radicals [mp.] OR Mitochondria [mp.] OR oxidative stress [mp.] OR reactive oxygen species [mp.] OR Nitric Oxide [mp.] OR nitric oxide synthase [mp.] OR lipid peroxidation [mp.] OR  reduced nicotinamide adenine dinucleotide phosphate oxidase [mp.] OR reactive nitrogen species [mp.] OR antioxidant [mp.] OR free radical scavenger [mp.] OR Free radicals [MeSH Terms] OR Mitochondria [MeSH Terms] OR oxidative stress [MeSH Terms] OR reactive oxygen species [MeSH Terms] OR Nitric Oxide [MeSH Terms] OR nitric oxide synthase [MeSH Terms] OR lipid peroxidation [MeSH Terms] OR  reduced nicotinamide adenine dinucleotide phosphate oxidase [MeSH Terms] OR reactive nitrogen species [MeSH Terms] OR antioxidant [MeSH Terms] OR scavenger [MeSH Terms]

OR

Excitatory amino acid [mp.] OR glutamate acid [mp.] OR excitatory amino acid transporter [mp.] OR neurotransmitter receptor [mp.] OR glutamate receptor [mp.] OR  n methyl dextro aspartic acid receptor [mp.] OR  AMPA receptor [mp.] OR Neurons [mp.] OR astrocytes [mp.] OR oligodendroglia [mp.] OR synapses [mp.] OR calcium signalling [mp.] OR Calcium[mp.] OR Excitatory amino acid [MeSH Terms] OR glutamate acid [MeSH Terms] OR excitatory amino acid transporter [MeSH Terms] OR neurotransmitter receptor [MeSH Terms] OR glutamate receptor [MeSH Terms] OR  n methyl dextro aspartic acid receptor [MeSH Terms] OR  AMPA receptor [MeSH Terms] OR Neurons [MeSH Terms] OR astrocytes [MeSH Terms] OR oligodendroglia [MeSH Terms] OR synapses [MeSH Terms] OR calcium signalling [MeSH Terms] OR Calcium[MeSH Terms]

OR

Inflammation [mp.] OR Inflammation Mediators [mp.] OR Inflammatory [mp.] OR neurogenic inflammation [mp.] OR Chemokines [mp.] OR Cytokines [mp.] OR RNA [mp.] OR MicroRNAs [mp.] OR complement [mp.] OR Leukocytes [mp.] OR Neutrophils [mp.] OR Microglia [mp.] OR interleukin [mp.] OR Inflammation [MeSH Terms] OR Inflammation Mediators [MeSH Terms] OR neurogenic inflammation [MeSH Terms] OR Chemokines [MeSH Terms] OR Cytokines [MeSH Terms] OR RNA [MeSH Terms] OR MicroRNAs [MeSH Terms] OR complement [MeSH Terms] OR Leukocyte [MeSH Terms] OR Neutrophil [MeSH Terms] OR Microglia [MeSH Terms] OR interleukin derivative [MeSH Terms]

**Web of Knowledge**

TS=(brain ischemia OR brain ischaemia OR brain ischemic OR brain infarctions OR brain infarction OR cerebral infarction OR cerebral infarctions OR stroke OR ischemic stroke)

AND

TS=(Apoptosis OR Necrosis OR Cell death OR caspases OR Enzyme activation OR Free radicals OR Mitochondria OR oxidative stress OR reactive oxygen species OR Nitric Oxide OR nitric oxide synthase OR lipid peroxidation OR NADPH oxidase OR reactive nitrogen species OR antioxidant OR free radical scavenger OR Excitatory amino acid OR glutamate acid OR excitatory amino acid transporter OR receptors, neurotransmitter OR receptors, glutamate OR receptors N-Methyl-D-Aspartate OR receptors, AMPA OR Neurons OR astrocytes OR oligodendroglia OR synapses OR Cortical spreading depression OR anoxia OR calcium signalling OR Calcium OR Inflammation OR Inflammation Mediators OR Inflammatory OR neurogenic inflammation OR Chemokines OR Cytokines OR RNA OR MicroRNAs OR complement system protein OR Leukocytes OR Neutrophils OR Microglia OR Interlukins)
