# Supplementary material for: Human Ischaemic Cascade Studies Using SH-SY5Y Cells: a Systematic Review and Meta-Analysis
Source: Transl Stroke Res. 2018 Mar 23;9(6):564–74. doi: 10.1007/s12975-018-0620-4 (PMC6208743; doi:10.1007/s12975-018-0620-4)
Supplement: Supplementary file 8 — (DOCX 14 kb) [file 12975_2018_620_MOESM8_ESM.docx]

**Pubmed**

SHSY5Y[All Fields] OR SH-SY5Y[All Fields] OR SH-SY-5Y[All Fields] OR SHSY-5Y[All Fields] OR SH-SY[All Fields] ->

**SH-SY(5)Y will be automatically interperated as SH-SY[All Fields] AND 5[All Fields] AND Y[All Fields]**

("brain ischaemia"[All Fields] OR "brain ischemia"[MeSH Terms] OR ("brain"[All Fields] AND "ischemia"[All Fields]) OR "brain ischemia"[All Fields]) OR (("brain"[MeSH Terms] OR "brain"[All Fields]) AND ("ischemia"[MeSH Terms] OR "ischemia"[All Fields] OR "ischemic"[All Fields]))) OR ("brain infarction"[MeSH Terms] OR ("brain"[All Fields] AND "infarction"[All Fields]) OR "brain infarction"[All Fields] OR ("brain"[All Fields] AND "infarctions"[All Fields]) OR "brain infarctions"[All Fields])) OR ("brain infarction"[MeSH Terms] OR ("brain"[All Fields] AND "infarction"[All Fields]) OR "brain infarction"[All Fields] OR ("brain"[All Fields] AND "infarctions"[All Fields]) OR "brain infarctions"[All Fields])) OR ("cerebral infarction"[MeSH Terms] OR ("cerebral"[All Fields] AND "infarction"[All Fields]) OR "cerebral infarction"[All Fields])) OR ("cerebral infarction"[MeSH Terms] OR ("cerebral"[All Fields] AND "infarction"[All Fields]) OR "cerebral infarction"[All Fields] OR ("cerebral"[All Fields] AND "infarctions"[All Fields]) OR "cerebral infarctions"[All Fields])) OR ("stroke"[MeSH Terms] OR "stroke"[All Fields])) OR (("ischemia"[MeSH Terms] OR "ischemia"[All Fields] OR "ischemic"[All Fields]) AND ("stroke"[MeSH Terms] OR "stroke"[All Fields]))) OR ("brain ischaemia"[All Fields] OR "brain ischemia"[MeSH Terms] OR ("brain"[All Fields] AND "ischemia"[All Fields]) OR "brain ischemia"[All Fields])) OR "brain ischemia"[MeSH Terms]) OR "stroke"[MeSH Terms]) OR "cerebral infarction"[MeSH Terms]->

**Web of knowledge:**

TS=(SHSY5Y OR SH-SY5Y OR SH-SY-5Y OR SHSY-5Y OR SH-SY(5)Y) -

TS=(brain ischemia OR brain ischaemia OR brain ischemic OR brain infarctions OR brain infarction OR cerebral infarction OR cerebral infarctions OR stroke OR ischemic stroke)->

**Embase**: all fields, OR,

(SHSY5Y or SH-SY5Y or SH-SY-5Y or SHSY-5Y or SH-SY).af. ->

(brain ischemia or brain ischaemia or brain ischemic or brain infarctions or brain infarction or cerebral infarction or cerebral infarctions or cerebrovascular accident).mp. [mp=title, abstract, heading word, drug trade name, original title, device manufacturer, drug manufacturer, device trade name, keyword, floating subheading]
